# Strategic Structural Control of Polyserotonin Nanoparticles and Their Application as pH-Responsive Nanomotors

**DOI:** 10.3390/nano14060519

**Published:** 2024-03-14

**Authors:** Junyi Hu, Jingjing Cao, Jinwei Lin, Leilei Xu

**Affiliations:** State Key Laboratory of Advanced Technology for Materials Synthesis and Processing, International School of Materials Science and Engineering, Wuhan University of Technology, Wuhan 430070, China

**Keywords:** polyserotonin nanoparticles, mesoporous, anisotropic, nanomotor, pH responsiveness

## Abstract

Serotonin-based nanomaterials have been positioned as promising contenders for constructing multifunctional biomedical nanoplatforms due to notable biocompatibility, advantageous charge properties, and chemical adaptability. The elaborately designed structure and morphology are significant for their applications as functional carriers. In this study, we fabricated anisotropic bowl-like mesoporous polyserotonin (PST) nanoparticles with a diameter of approximately 170 nm through nano-emulsion polymerization, employing P123/F127 as a dual-soft template and 1,3,5-trimethylbenzene (TMB) as both pore expander and emulsion template. Their formation can be attributed to the synchronized assembly of P123/F127/TMB, along with the concurrent manifestation of anisotropic nucleation and growth on the TMB emulsion droplet surface. Meanwhile, the morphology of PST nanoparticles can be regulated from sphere- to bowl-like, with a particle size distribution ranging from 432 nm to 100 nm, experiencing a transformation from a dendritic, cylindrical open mesoporous structure to an approximately non-porous structure by altering the reaction parameters. The well-defined mesopores, intrinsic asymmetry, and pH-dependent charge reversal characteristics enable the as-prepared mesoporous bowl-like PST nanoparticles’ potential for constructing responsive biomedical nanomotors through incorporating some catalytic functional materials, 3.5 nm CeO_2_ nanoenzymes, as a demonstration. The constructed nanomotors demonstrate remarkable autonomous movement capabilities under physiological H_2_O_2_ concentrations, even at an extremely low concentration of 0.05 mM, showcasing the 51.58 body length/s velocity. Furthermore, they can also respond to physiological pH values ranging from 4.4 to 7.4, exhibiting reduced mobility with increasing pH. This charge reversal-based responsive nanomotor design utilizing PST nanoparticles holds great promise for advancing the application of nanomotors within complex biological systems.

## 1. Introduction

The diminutive size, expansive surface area, and diverse functional attributes of micro/nanomotors (MNMs) confer a broad spectrum of applications and manipulation capabilities within microenvironmental interactions, nano-bio-interfaces, and cellular contexts [1,2,3,4,5]. Particularly in the biomedical field, intelligent MNMs have the potential to revolutionize drug delivery, cancer treatment, and in vivo imaging [6,7,8]. Designing and synthesizing responsive MNMs are effective strategies to achieve their intelligent functionalities. Notably, the utilization of pH diversity within organisms as a characteristic signal for biorecognition has garnered substantial attention, leading to the prominence of pH-responsive MNMs [9,10,11]. The current paradigm of smart responsiveness involves MNMs autonomously reacting to external stimuli or environmental changes. Among these, harnessing the pH gradient in the physiological milieu for navigation and motion control of MNMs represents a novel and intelligent approach [12,13,14,15,16]. This mainly involves the modification of pH-responsive media such as polymers or catalytic materials with pH-dependent activities on the MNM surface, and the motion direction and enhancement are predominantly achieved through alterations in the reactions of pH-responsive media and substrate [17,18,19]. However, while loading pH-responsive media onto nanomotors enhances their adaptability to varying pH environments, it tends to occupy active sites on the nanomotor surface, potentially impeding the loading of fuel by MNMs. Additionally, the introduction of a pH-responsive medium may complicate the structure to some extent and pose potential challenges in terms of biocompatibility. Consequently, the development of carriers with inherent responsiveness emerges as a solution.

Serotonin, a neurotransmitter and biogenic amine ubiquitous in living organisms, is a type of bioactive molecule possessing a molecular structure akin to dopamine [20,21,22,23,24]. Consequently, serotonin-based nanoparticles (polyserotonin, PST) share biochemical properties quite similar to polydopamine involving biocompatibility, photothermal properties, and easy functionalization [25,26]. In addition, it exhibits a distinctive attribute of pH-regulated surface charge due to the protonation of surface amine groups under acidic conditions leading to an increase in surface positive charges. This enables PST nanoparticles to possess pH-dependent degradation properties and drug delivery capacities, conferring distinct advantages for biomedical applications. However, current research on PST nanoparticles is still in its infancy with monodisperse nanoparticles primarily achieved by self-polymerization of serotonin monomer molecules under basic conditions [27,28]. This restricts its further application, especially serving as a functional carrier.

In this study, anisotropic bowl-like mesoporous PST nanoparticles were elaborately fabricated by an interface-directed emulsion polymerization approach, inspired by the synthesis of polydopamine (PDA) [29,30,31]. The monomer, serotonin, was utilized alongside P123/F127/TMB, which formed stable composite micelles acting as nucleation sites. The serotonin monomer was further self-assembled on these composite micelles under alkaline conditions. Precise control over the soft stencil ratios of P123/F127 facilitated the formation of micelles with different sizes, driven by weakly interacting van der Waals forces between P123, F127, and TMB. This resulted in a series of pore structure transformations, transitioning from dendritic spherical structures to mesoporous bowl-like configurations. Utilizing mesoporous bowl-shaped PST as a carrier, CeO_2_ nanoenzymes were electrostatically adsorbed onto the carrier’s surface, capitalizing on its low symmetry and large opening structure. The resulting CeO_2_@PST nanomotors enhanced diffusive motion at the physiological H_2_O_2_ concentrations. Furthermore, their unique charge reversal property endows the CeO_2_@PST nanomotors with the property of regulating surface hydrophilic-hydrophobic states, thus enabling adaptable motion in the physiological pH regions. The utilization of carriers possessing variable surface charges presents a promising approach, providing an alternative strategy for the fabrication of nanomotors capable of adaptable motion in complex physiological microenvironments.

## 2. Materials and Methods

### 2.1. Materials

Serotonin hydrochloride (5-HT), Pluronic P123 and F127, 1,3,5-trimethyl benzene (TMB), ammonia solution (25–28%), tetramethylammonium hydroxide (TMAOH), Ce(NO_3_)_3_•6H_2_O, and sodium citrate were purchased from Sigma-Aldrich (St. Louis, MO, USA). The catalase assay kit was purchased from Beyotime Biotechnology. Ethanol and hydrogen peroxide were purchased from TCI (Shanghai) Development Co., Ltd., Shanghai, China. All reagents were used without further purification. Deionized (DI) water was used in all experiments.

### 2.2. Synthesis of PST Nanoparticles

The PST with a bowl-shaped structure was synthesized based on the dual-soft-template method. In a typical synthesis, P123 (25 mg), F127 (75 mg), 5-HT (150 mg), and TMB (0.8 mL) were mixed in the solvent of deionized water (7.5 mL) and ethanol (2.5 mL). The mixture was formed in an emulsion solution by ultrasonication (360 W) for 10 min. Then, ammonia (0.6 mL) was quickly added to the emulsion solution. After 18 h of magnetic stirring (600 r/min), the product of PST was collected by centrifugation (10,000 r/min) and then washed with water several times. Finally, it was dried in an oven at 60 °C.

### 2.3. Synthesis of CeO_2_ Nanoenzymes

In a standard methodology, a solution of TMAOH (50 mL, 50 mM) was introduced into a solution containing Ce(NO_3_)_3_•6H_2_O (0.434 g) and sodium citrate (0.516 g) within a 250 mL glass bottle. The resulting mixture underwent stirring (600 r/min) at room temperature (RT) for 21 h. Subsequently, the reaction mixture was transferred to a three-necked round-bottom flask and subjected to reflux at 100 °C for a duration of 4 h. The product was collected by centrifugation (10,000 r/min) for 20 min.

### 2.4. Synthesis of CeO_2_@PST Nanomotors

The PST (1 mg) was completely dispersed in a phosphate buffer solution (PBS) at pH 6.5, followed by the addition of CeO_2_ (1 mg). Magnetic stirring (600 r/min) was conducted for 24 h at room temperature to obtain the CeO_2_@PST nanomotors, which were further isolated through a process of differential centrifugation (8000 r/min), ensuring thorough washing with pure water.

### 2.5. Motion Tests of the CeO_2_@PST Nanomotors

The nanomotors’ motion was studied in solutions with varying concentrations of H_2_O_2_ or at different pH levels of the H_2_O_2_ solution. For each trial, the motors were dispersed in an aqueous solution and placed on a glass slide. Subsequently, the nanomotors suspended in the H_2_O_2_ solution were deposited onto the droplet for observation. Recordings were made using an optical microscope equipped with a high-resolution charge-coupled device (CCD) digital camera.

### 2.6. Characterizations

Transmission electron microscopy (TEM) and energy-dispersive X-ray (EDX) images were acquired using an FEI F200 microscope from the United States. Dynamic light scattering (DLS) measurements were performed utilizing a NanoBrook 90Plus Zeta instrument (San Jose, CA, USA). Motion videos and images were captured employing a Leica DM 3000B optical microscope sourced from Germany. Fourier transform infrared (FT-IR) spectroscopy analyses were carried out on a NEXUS 670 FTIR spectrometer within the range of 400 cm^−1^ to 4000 cm^−1^ using KBr pellets. X-ray photoelectron spectroscopy (XPS) measurements were conducted utilizing an ESCALAB MKII photoelectron spectrometer equipped with a standard Al anode. N_2_ adsorption–desorption isotherms were generated using a Micromeritics ASAP 2020M automated sorption analyzer. Contact angles (CA) were measured employing the optical contact angle measuring instrument DSA100S from Hamburg, Germany, manufactured by KRUSS.

## 3. Results and Discussion

### 3.1. Preparation and Characterization of Anisotropic Mesoporous Bowl-like PST Nanoparticles

**Figure 1:**
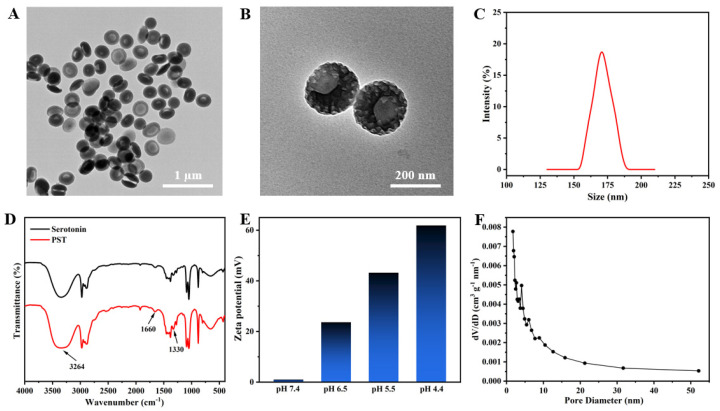
(**A**) Low and (**B**) high magnification TEM images, and (**C**) the particle size distribution of bowl-like PST nanoparticles. (**D**) FTIR spectra of serotonin monomers and PST nanoparticles. (**E**) Zeta potentials of PST at different pH conditions. (**F**) Pore size distribution curve of bowl-like PST nanoparticles.

The fabrication of mesoporous bowl-like PST nanoparticles involved using serotonin as the monomer seed, P123/F127 as a dual-soft-template, and TMB as both a pore expander and an emulsion template. TEM images reveal a mesoporous bowl-like structure with uniform morphology at low magnification (Figure 1A), showcasing a prominent open structure at higher magnification, adorned with conspicuous columnar holes on the surface (Figure 1B). The hydrated particle size of the as-prepared PST nanoparticles was approximately 170 nm (Figure 1C). In Figure 1D, the FTIR spectra of serotonin monomer and PST exhibit absorption peaks at 1330 cm^−1^ for the representative amine groups of serotonins. The analysis also shows intermolecular hydrogen bonding of the phenolic hydroxyl group of the benzene ring, shifting to a lower wavenumber at 3264 cm^−1^. Simultaneously, the peak of the C=C double bond of the benzene ring at 1660 cm^−1^ increased in intensity, indicating the polymerization of serotonin from monomer to polyserotonin. The zeta potential in Figure 1E of PST is scrutinized under physiological pH conditions, showing near neutrality at pH 7.4. The gradual positive charge enhancement occurs with decreasing pH, reaching +60 mV at pH 4.4, signifying surface amine group protonation and positive surface charge acquisition under acidic conditions. The corresponding pore size distribution exhibits multiple peaks between 2 nm and 7 nm, affirming the mesoporous nature of PST (Figure 1F). The specific surface area measures approximately 66.13 m^2^/g. The characterization of representative samples confirms the successful synthesis of anisotropic bowl-shaped mesoporous PST nanoparticles.

### 3.2. Structural Evolution and Formation Mechanism of Anisotropic Mesoporous Bowl-like PST Nanoparticles

**Figure 2:**
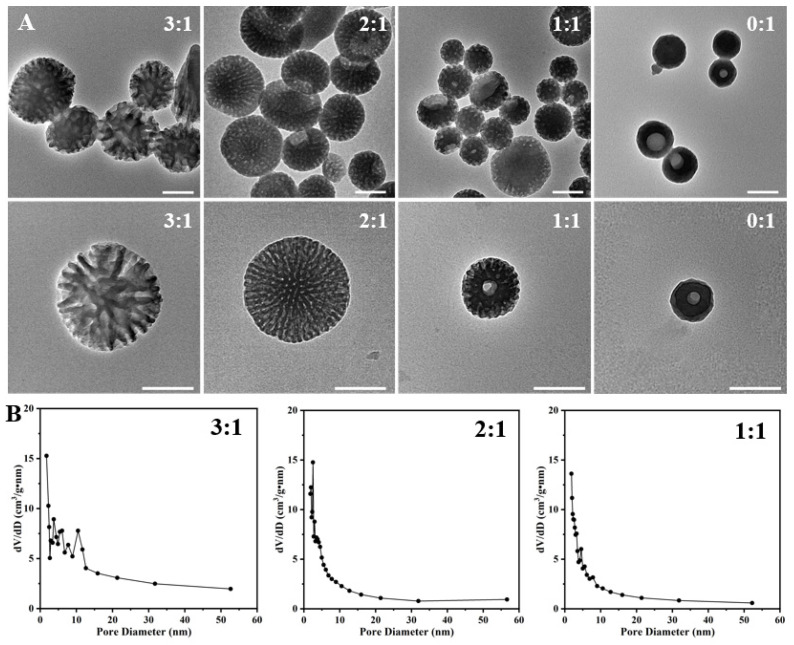
(**A**) Typical TEM images of the as-prepared PST nanoparticles with varying P123/F127 weight ratios of 3/1, 2/1, 1/1, and 0/1. (**B**) Pore size distribution curves of the as-prepared PST nanoparticles with different P123/F127 weight ratios. Bar = 200 nm.

In order to find out the formation mechanism, we first studied the effect of the weight ratio of P123 and F127 on the structure evolution of the as-prepared PST nanoparticles (Figure 2A,B and Figure S1). As depicted in the TEM images, adopting varying P123/F127 ratios yields distinct morphological features in the mesopores of the prepared materials. When the ratio is maintained at 3:1, a distinct spherical configuration with dendritic mesopores is observed, characterized by a particle size of approximately 432 nm. The surface area is determined to be 53.59 m^2^/g, with the predominant pore size distribution falling within the range of 3 nm to 10 nm. Subsequently, at a ratio of 2:1, a transformation occurs towards a more mesoporous spherical structure with a partial bowl structure, accompanied by a reduction in pore size with peaks observed at 2.5 nm. Correspondingly, the surface area diminishes significantly to 41.58 m^2^/g. A further reduction in the ratio to 1:1 results in a noteworthy reduction in particle size, manifesting a mesoporous bowl-like structure and spherical structure with a diameter of approximately 221 nm. The pore size distribution exhibits peaks at 2.5 nm, 4.5 nm, and 7.8 nm, accompanied by a surface area reduction to 39.72 m^2^/g. In the absence of P123 incorporation during the synthesis procedure, a nearly non-porous bowl-like configuration was obtained, accompanied by an unattainable characterization of pore size distribution. The particle size is decreased to be approximately 100 nm. These results indicate that the weight ratio of P123 and F127 greatly impacts the assembly process of serotonin, thereby determining the structure of the as-prepared PST nanoparticles.

**Figure 3:**
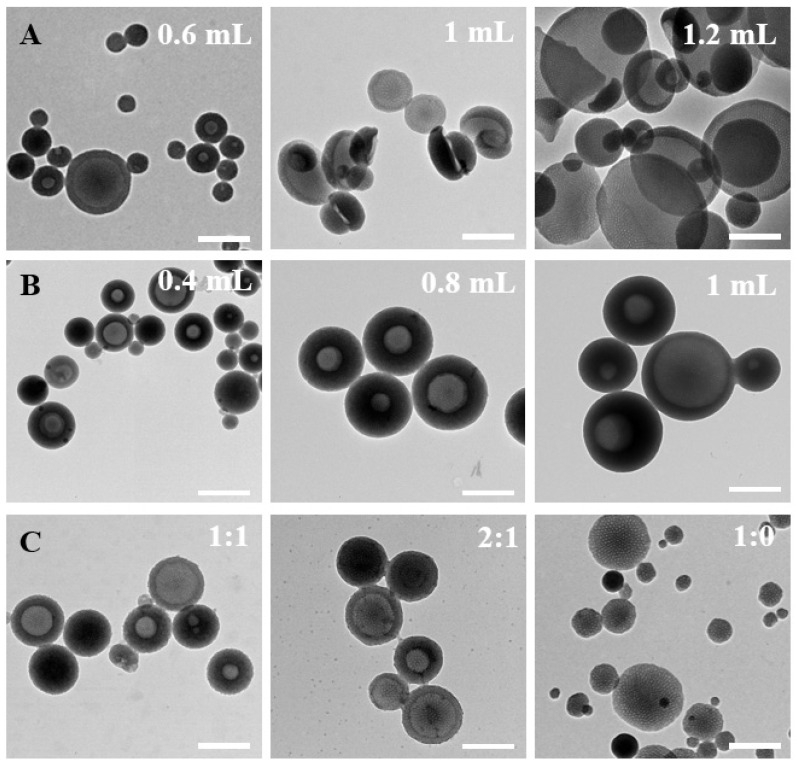
Representative TEM images of the as-prepared PST nanoparticles obtained with different (**A**) TMB amounts of 0.6 mL, 1 mL, and 1.2 mL, (**B**) ammonia amounts of 0.4 mL, 0.8 mL, and 1 mL, and (**C**) water/ethanol volume ratios of 1:1, 2:1, and 1:0. Bar = 500 nm.

In addition, the inquiry into the formation mechanism was also conducted through meticulous control of various factors throughout the synthesis process. Initially, the impact of TMB amounts on particle formation was systematically investigated (Figure 3A). A noteworthy increase in the mesoporous bowl structure’s opening size was observed with increasing the amount of TMB. Beyond 1.2 mL of TMB addition, a distinctive 2D plate structure emerged. During the preparation process, TMB exhibited dual functionality by contributing to the formation of surface mesoporous pores and acting as an emulsion template for the formation of bowl-like structures. The increased addition of TMB facilitated TMB droplets to function as templates, inducing micelle growth on the TMB droplets while concurrently forming composite micelles. Subsequently, the influence of ammonia amounts on the formation of bowl-shaped nanoparticles was scrutinized (Figure 3B). The quantity of ammonia significantly impacted both the uniformity and size of the particles. Ammonia functioned as a catalyst, influencing the rate of serotonin monomer polymerization. Optimal uniformity, with particle sizes below 200 nm, was achieved when 0.6 mL of ammonia was added. This underscored the stringent requirement of an alkaline environment for the polymerization of serotonin monomers. The exploration extended to solvent ratios, with controlled water/ethanol ratios of 1:1, 2:1, 3:1, and 1:0 (Figure 3C). The solvent ratios demonstrated a discernible effect on both particle size and uniformity. Particularly, at a solvent ratio of 3:1, the particles exhibited a substantial decrease in size, indicating heightened stability in this configuration. In the absence of ethanol as a solvent, the formation of the mesoporous bowl structure was precluded, and the resultant particle size exhibited marked non-uniformity. This emphasizes that ethanol addition facilitated the expansion of the hydrophobic volume within composite micelles, fostering a more stable micelle structure. The adept manipulation of ethanol proportion was revealed as an effective strategy to control the particle size and the formation of mesopores.

In view of the above results, the formation mechanism of anisotropic bowl-like mesoporous PST nanoparticles was proposed. The interplay between TMB and soft templates generated a microemulsion system containing composite micelles (P123/F127/TMB) through van der Waals forces. Monomer seeds interacted with TMB on the micelle surface through π-π stacking forming pore structures. Subsequently, utilizing TMB droplets as emulsion templates, composite micelles undergo island-like growth on the surface of TMB droplets, thereby forming a bowl-shaped structure. The P123/F127/TMB amalgamation orchestrates the assembly of micelles at the interfaces, culminating in the simultaneous self-assembly and polymerization of monomer seeds on the micelles. The reaction begins with the oxidation of serotonin to serotonin-quinone, which then undergoes branching reactions to produce cross-linked polymers, forming PST nanoparticles [25]. P123 and F127 assume roles as soft templates, actively engaging in the formation of composite micelles and exerting influence over their size. P123 and F127, both amphiphilic polymers comprising hydrophilic polyethylene oxide (PEO) chains and hydrophobic polypropylene oxide (PPO) chains, differ in the length of their hydrophilic chains, with P123 exhibiting shorter chains and a smaller molecular weight. With a fixed total mass of P123 and F127, an elevated proportion of P123 corresponds to a greater number of participating molecules in composite micelle formation. The resulting larger micelles accommodate a higher TMB content, diminishing the liquid template’s TMB concentration and fostering the creation of three-dimensional spherical structures with augmented particle sizes. Conversely, decreasing the proportion of P123 reduces the number of involved molecules, resulting in smaller composite micelles. The surplus TMB in the system contributes to the formation of emulsion templates, serving as interfaces for co-assembly with composite micelles. This collaborative effort engenders bowl-like particles characterized by diminished mesoporous apertures. Notably, the augmentation of P123 enhances the surfactant’s filling parameter, harmonizing the effective micelle assembly in the emulsion and thereby steering the structural evolution of mesoporous PST nanoparticles. The observed variability in products attests to the substantive impact of soft template properties on mesoscopic structures.

### 3.3. Synthesis and Characterization of CeO_2_@PST Nanomotors

Good biocompatibility, well-defined mesopores, intrinsic asymmetry, and a unique pH-dependent charge reversal characteristic enable the anisotropic mesoporous bowl-like PST nanoparticles to serve as an excellent carrier for constructing responsive nanomotors. CeO_2_ nanoenzymes, with a well-dispersed particle size of approximately 3.5 nm, were incorporated into the as-prepared PST nanoparticles through electrostatic attraction. Their catalase (CAT)-like catalytic activity was employed to provide driving forces for the obtained nanomotors. The morphology and composition of the CeO_2_ nanoenzymes were characterized by TEM, DLS, and XPS, respectively, as shown in Figures S2–S4. TEM and DLS characterizations reveal that CeO_2_ nanoenzymes exhibit a spherical morphology with uniform particle size and good dispersion. XPS analysis elucidates the valence state composition of surface cerium ions, indicating the coexistence of Ce^3+^ and Ce^4+^ states on the surface. This suggests that the CeO_2_ nanoenzymes possess CAT-like catalytic activity. Their CAT-like activity was assessed through the Michaelis–Menten constants (K_m_) value of 32.75 mM, indicating robust catalytic efficacy (Figures S5 and S6).

**Figure 4:**
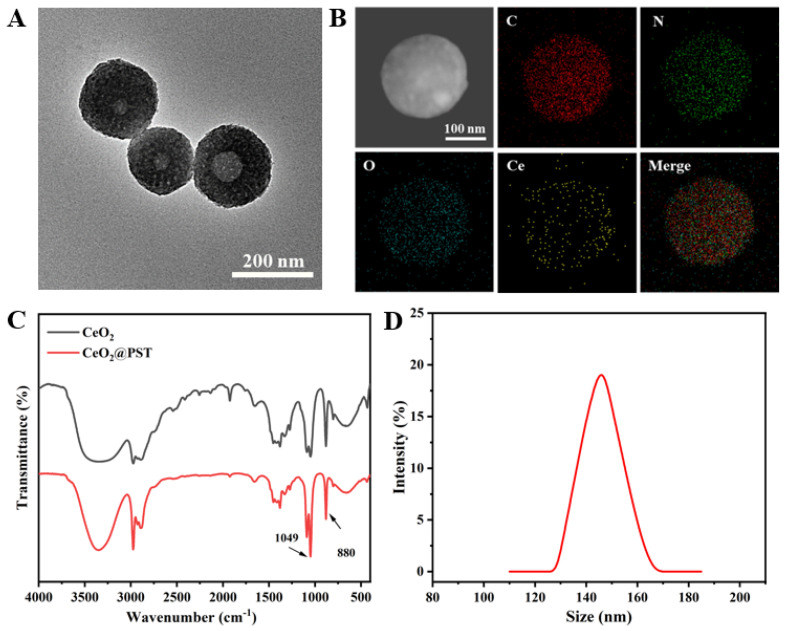
(**A**) TEM image and (**B**) element mapping images of the CeO_2_@PST nanomotors. (**C**) FTIR spectra of the CeO_2_ nanoenzymes and the CeO_2_@PST nanomotors. (**D**) The particle size distribution curve of the CeO_2_@PST nanomotors.

The morphology of the constructed nanomotors (CeO_2_@PST) was characterized by TEM. As shown in Figure 4A, a perceptible roughening surface and a partially filled porous structure were observed compared to that of PST nanocarriers. The infrared peaks of CeO_2_@PST show the presence of characteristic peaks of PST at 1330 cm^−1^ and 1660 cm^−1^ in its backbone. Combined with the Ce elemental analysis obtained from EDS mapping, it can be concluded that CeO_2_ nanoenzymes are uniformly distributed in CeO_2_@PST nanomotors (Figure 4B). The enhanced Ce-O lattice stretching and bending vibrations at 1049 cm^−1^ and 880 cm^−1^ further attest to the effective CeO_2_ loading (Figure 4C). The reduction in particle size compared to the PST carriers from 170 nm to 146 nm could be attributed to the degradation of PST during the weak acidic process of nanoenzyme modification (Figure 4D).

### 3.4. Autonomous Movement of CeO_2_@PST Nanomotors at the Physiological H_2_O_2_ Concentrations

Hydrogen peroxide (H_2_O_2_) is a widely employed fuel that possesses the capacity to propel MNMs. The driving force typically arises from its asymmetrically catalytic decomposition on the surface of MNMs, generating an asymmetric oxygen concentration gradient. This induces the surficial osmotic flow to drive the motion of MNMs. However, despite H_2_O_2_ being an endogenous substance in organisms within a concentration range from a few micromoles to dozens or even a hundred micromoles in tumor microenvironments [32,33,34], the millimole concentrations required by many MNM systems can give rise to biological toxicity, limiting their applications, particularly in biomedicine. Consequently, developing MNMs that can be propelled at a concentration below 100 µM is significant for their further applications.

**Figure 5:**
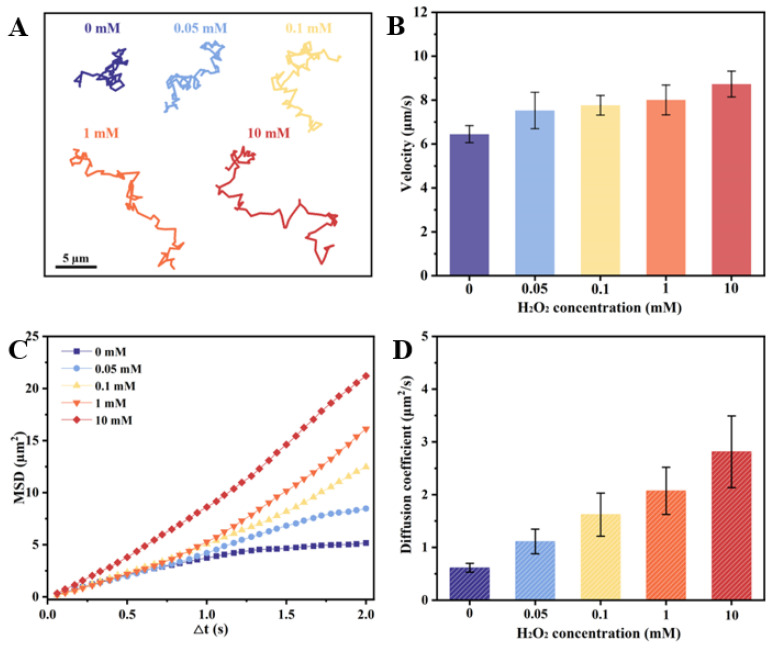
Motion behaviors of the CeO_2_@PST nanomotors at different H_2_O_2_ concentrations of (**A**) time-lapse trajectories in 5 s, (**B**) average moving velocities, (**C**) MSD values, and (**D**) diffusion coefficients.

The Inherent structural asymmetry of the as-prepared CeO_2_@PST nanomotors, resulting from their bowl-like carriers, induces asymmetric oxygen generation and establishes an oxygen concentration gradient across the particles, thereby contributing to their autonomous motion. The effect of H_2_O_2_ concentrations on their motion behaviors is shown in Figure 5. Comparative analysis with H_2_O_2_-free conditions reveals distinctive diffusion patterns in the nanomotor trajectories at 5 s (Figure 5A), indicating a notable increase in diffusion with elevated H_2_O_2_ concentrations. This enhanced diffusion property aligns consistently with motion velocity analysis, mean squared displacement (MSD), and diffusion coefficient assessments (Figure 5B–D). Thus, the incorporation of CeO_2_ nanoenzymes onto the asymmetric bowl-shaped PST carriers imparts the nanomotor with the requisite propulsive force for autonomous movement. In the context of the physiological microenvironment, where H_2_O_2_ concentrations vary in accordance with the organism’s inherent physiological nature, levels can range to 0.1 mM in disease microenvironments. Notably, at a concentration of 0.1 mM H_2_O_2_, the nanomotors demonstrate a remarkable autonomous speed of 7.766 µm/s, calculated as 51.58 body length/s velocity, accompanied by an enhanced diffusion coefficient of 1.622 µm^2^/s. This substantiates the capability of nanomotors to autonomous movement in response to the physiological concentrations of H_2_O_2_. Meanwhile, along with the increase in fuel concentration, the nanomotors exhibit enhanced diffusive motility, suggesting their fuel-dependent motility.

### 3.5. Responsive Motion Behaviors of CeO_2_@PST Nanomotors in the Physiological pH Regions

**Figure 6:**
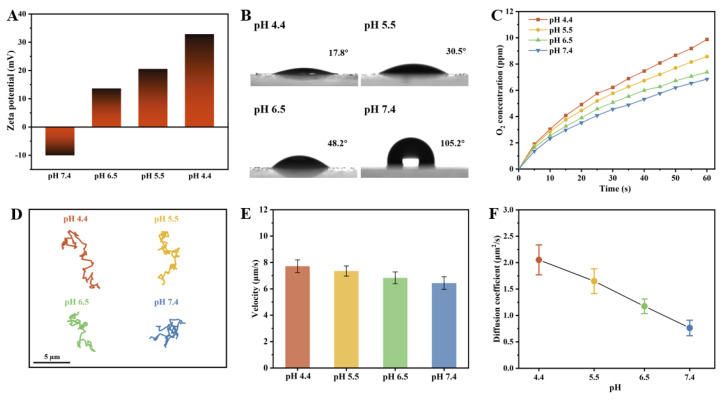
(**A**) Zeta potentials, (**B**) contact angles, and (**C**) the rate curves of oxygen generation per unit time of the CeO_2_@PST nanomotors at different pH conditions. Motion behaviors of the CeO_2_@PST nanomotors at different pH conditions, characterized by (**D**) time-lapse trajectories in 5 s, (**E**) average moving velocities, and (**F**) diffusion coefficients.

The pH value in physiological environments is variable and commonly utilized as a signal cue for the design of intelligent responsive delivery systems. For instance, the tumor microenvironment exhibits acidity (pH 6.5–6.8) lower than that of normal tissues (pH 7.15–7.45), while the pH of endosomes/lysosomes is even lower (pH 4.5–5.0) [35,36]. Benefiting from the pH-dependent charge reversal characteristics of the PST carriers, the as-prepared CeO_2_@PST nanomotors potentially exhibit pH responsiveness. The investigation of their responsiveness to pH values ranging from 4.4 to 7.4 contributes to advancing their potential applications. Upon incorporating surface-negatively charged CeO_2_ nanoenzymes, the surface positive charges in PST carriers are partially neutralized. Consequently, the CeO_2_@PST nanomotors display a negative zeta potential (−10 mV) at pH 7.4, escalating to +35 mV at pH 4.4 (Figure 6A). This observation indicates that the CeO_2_@PST nanomotors exhibit pH-dependent charge reversal characteristics. In general, the heightened positive surface charges induce an increase in surface energy, effectively attracting the negatively charged water molecules. This augmentation in mutual attraction facilitates enhanced water spreading on the surface, resulting in a reduced water contact angle. Evaluation of these properties through contact angle testing reveals hydrophobicity on the nanomotor surface at pH 7.4, evidenced by a contact angle of 105.2°. As the pH decreased, the surface transitioned to a significantly hydrophilic state at pH 4.4, with the contact angle decreasing to 17.8° (Figure 6B). The surface hydrophilic–hydrophobic state can greatly impact the interaction between nanoenzymes loaded on the nanomotor surface and the catalytic substrate, which is evidenced by the oxygen generation curves shown in Figure 6C. The as-prepared CeO_2_@PST nanomotors exhibit the highest hydrophilicity at pH 4.4, thereby demonstrating the fastest oxygen generation. As the pH value increases, a transmission from hydrophilicity to hydrophobicity leads to a decrease in oxygen generation. This undoubtedly influences the propulsion of the nanomotors.

The motion behaviors of the as-prepared CeO_2_@PST nanomotors were investigated under varying pH values within the physiological range. As shown in the trajectory graphs, the nanomotors display a significantly expanded displacement at acidic pH values, indicating their enhanced diffusion (Figure 6D). With an increase in pH from 4.4 to 7.4, a distinct reduction in mobility is characterized by the narrowed trajectory range, the decreased velocity, and the diffusion coefficient, underscoring the substantial impact of surface charge variations on their motion behaviors (Figure 6E,F). This trend is consistently reflected in the MSD curve (Figure S7). Evaluation of motion velocity and diffusion coefficient at different pH levels reveals a significant increase of 1.279 µm/s and 1.288 µm^2^/s, respectively, at pH 4.4 compared to pH 7.4.

The observed responsive motor behavior in diverse pH environments may be attributed to alterations in surface properties, specifically changes in surface charge influencing hydrophilic and hydrophobic characteristics. Under acidic conditions, the enhanced hydrophilic state facilitates efficient contact between nanoenzymes and catalytic substrates, resulting in heightened reaction substrates, increased catalytic product generation, amplified driving forces, and a conspicuous enhancement in diffusive movement. This aligns with the concept that increased catalytic product generation corresponds to greater driving forces. For CeO_2_@PST under different pH conditions, TEM and FTIR characterization were carried out, both of which corroborated that only the surface charge of the particles changed under the conditions of pH change (Figures S8 and S9). Essentially, the pH-responsive motion behavior of the nanomotor intricately links to the dynamic shift in surface charge properties. The inversion of surface charge can effectively dictate the nanomotor’s transition to distinct motion behaviors within disparate pH environments, thereby enhancing the adaptability of the nanomotor’s motion in complex settings.

## 4. Conclusions

In this study, we present a structure-controlled strategy for fabricating anisotropic mesoporous bowl-like PST nanoparticles and reveal their formation mechanism based on dual-soft template composite micelle assembly and simultaneous interface-induced anisotropic growth. In the preparation, TMB acts as both the pore swelling agent and the emulsion template. Its amount along with the weight ratio of P123 and F127 is essential for obtaining the anisotropic mesoporous bowl-like structure. Through the manipulation of the reaction conditions, we successfully transform the morphology of PST from a dendritic mesoporous spherical, cylindrical mesoporous bowl-like structure to an approximately non-porous bowl-like structure. The as-prepared anisotropic mesoporous bowl-like PST nanoparticles were employed to construct nanomotors by incorporating CeO_2_ nanoenzymes. The as-prepared nanomotors exhibit autonomous motion behaviors under physiological H_2_O_2_ concentrations relying on their instinct asymmetry of the bowl-like structure and the CAT-like catalytic activity. They also demonstrate pH-responsive motion behavior deriving from the pH-dependent charge reversal characteristic of the PST nanoparticles, which influences the surface hydrophilicity of the nanomotors. The employment of the carriers with environment-dependent surface charges offers an alternative approach for constructing intelligent MNMs capable of adapting to diverse delivery scenarios, further promoting their potential applications.

## Data Availability

Data are contained within the article.

## Supplementary Materials

The following supporting information can be downloaded at https://www.mdpi.com/article/10.3390/nano14060519/s1, Figure S1. N_2_ adsorption/desorption isotherms of PST nanoparticles at different P123/F127 mass ratios. Figure S2. TEM images of CeO_2_ nanoenzymes. Figure S3. The particle size distribution curve of CeO_2_ nanoenzymes. Figure S4. X-ray photoelectron spectroscopy (XPS) analysis of the valence composition of cerium ions on CeO_2_ nanoenzymes. Figure S5. The Lineweaver-Burk linear fitting for CeO_2_ CAT-like enzymatic activity analysis. Figure S6. The activity curve of CeO_2_ nanoenzymes. Figure S7. MSD values of the CeO_2_@PST nanomotors moving under different pH conditions. Figure S8. TEM images of CeO_2_@PST nanomotors at different pH conditions. Figure S9. FTIR spectra of the CeO_2_@PST nanomotors at different pH conditions.
