# Supplementary material for: Strategic Structural Control of Polyserotonin Nanoparticles and Their Application as pH-Responsive Nanomotors
Source: Nanomaterials (Basel). 2024 Mar 14;14(6):519. doi: 10.3390/nano14060519 (PMC10975037; doi:10.3390/nano14060519)
Supplement: Supplementary file 1 [file nanomaterials-14-00519-s001.zip › nanomaterials-2901366-supplementary.pdf]

nanomaterials

Supplementary Materials

Strategic Structural Control of Polyserotonin Nanoparticles and Their Application as pH-Responsive Nanomotors

Junyi Hu, Jingjing Cao, Jinwei Lin and Leilei Xu *

State Key Laboratory of Advanced Technology for Materials Synthesis and Processing, International School of Materials Science and Engineering, Wuhan University of Technology, Wuhan 430070, China

* Correspondence: L. X. (xull@whut.edu.cn)

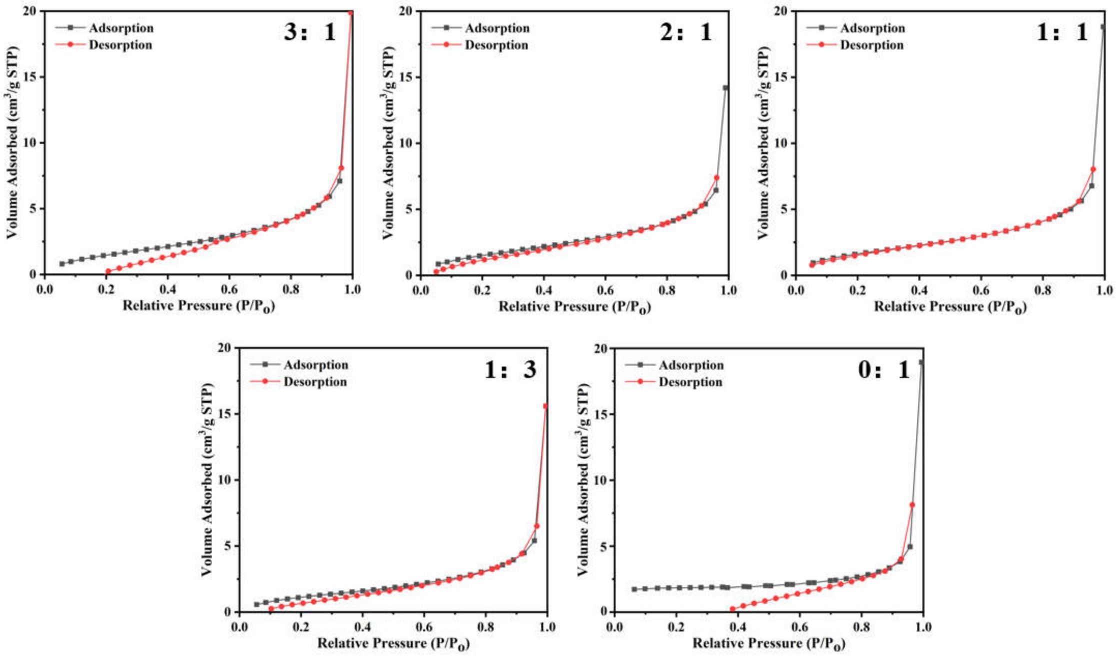

Figure S1. N_2 adsorption/desorption isotherms of PST nanoparticles at different P123/F127 mass ratio.

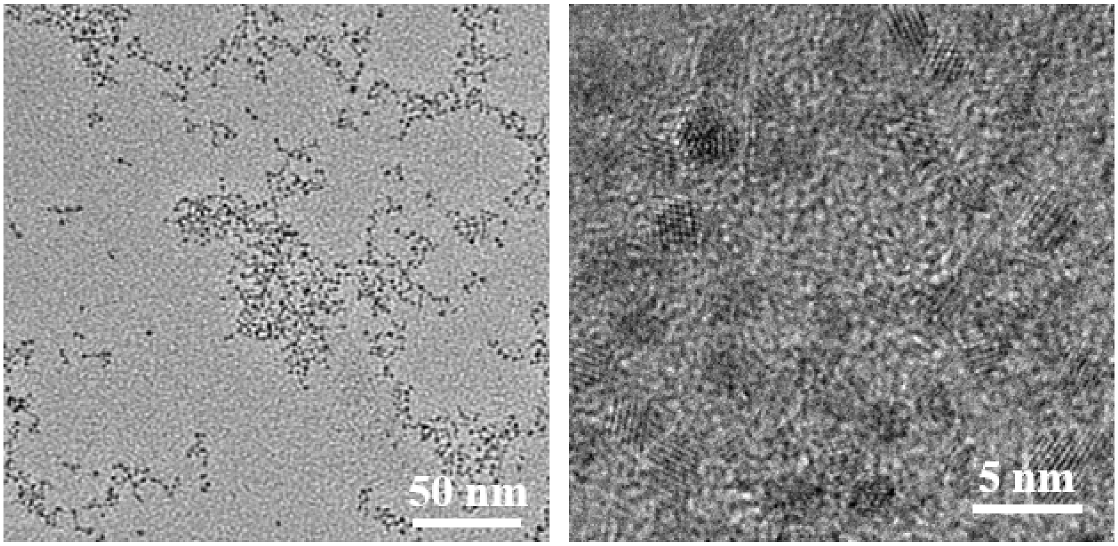

Figure S2. TEM images of CeO_2 nanoenzymes.

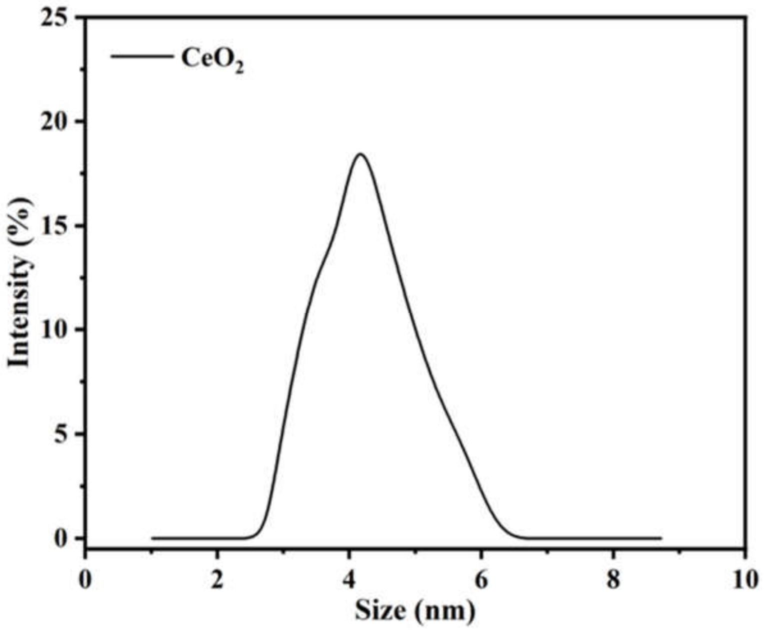

Figure S3. The particle size distribution curve of CeO_2 nanoenzymes.

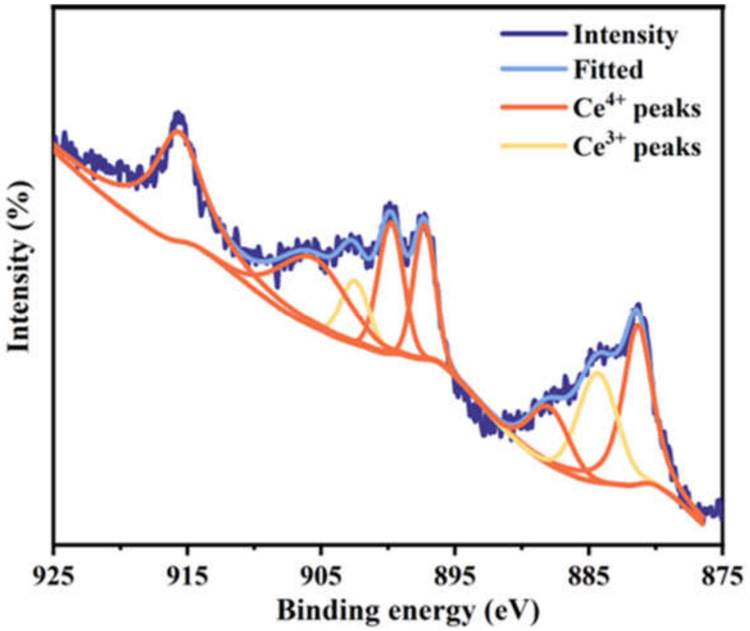

Figure S4. X-ray photoelectron spectroscopy (XPS) analysis of the valence composition of cerium ions on CeO_2 nanoenzymes.

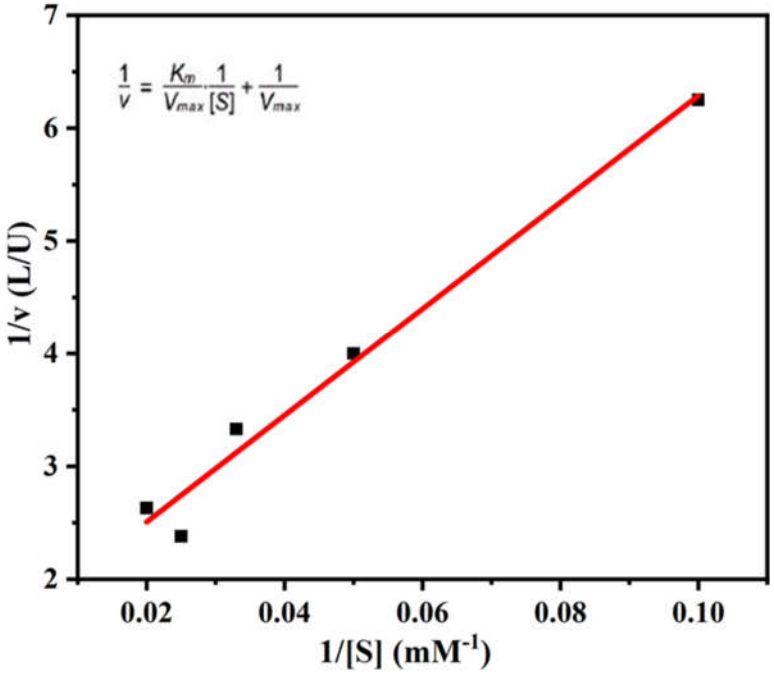

Figure S5. The lineweaver-Burk linear fitting for CeO_2 CAT-like enzymatic activity analysis.

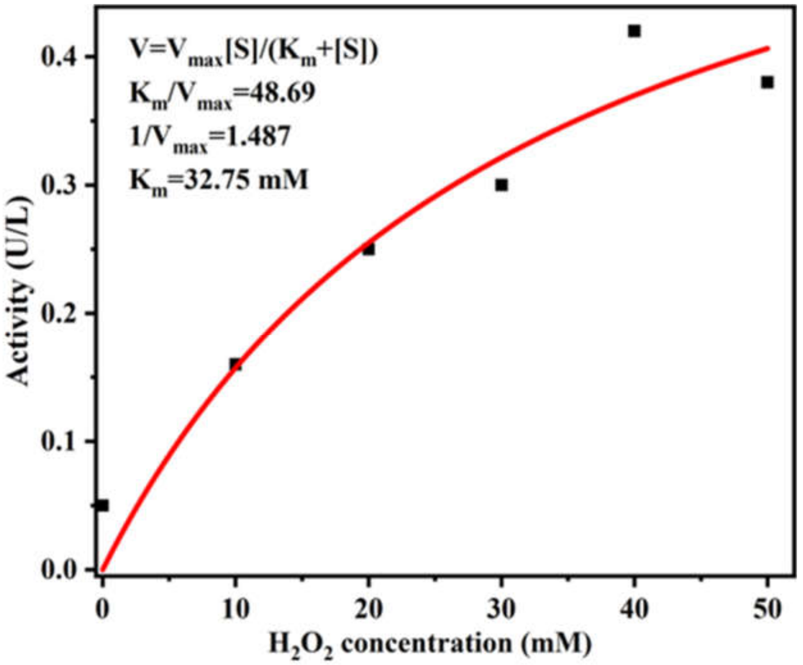

Figure S6. The activity curve of CeO_2 nanoenzymes.

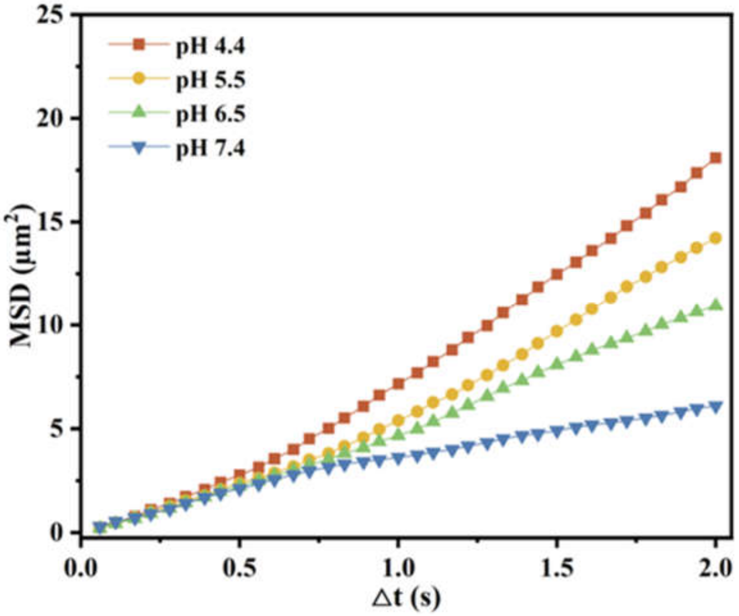

Figure S7. MSD values of the CeO_2@PST nanomotors moving under different pH conditions.

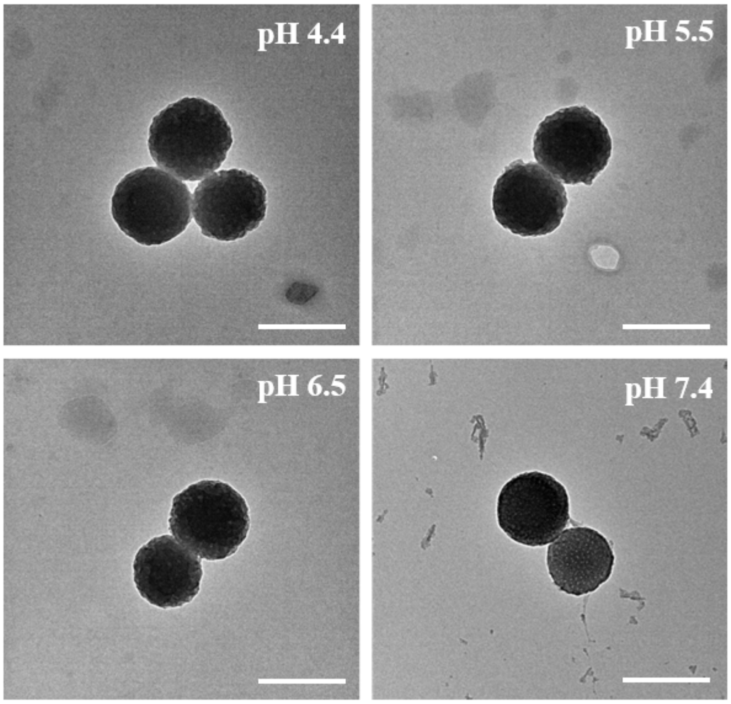

Figure S8. TEM images of CeO_2@PST nanomotors at different pH conditions.

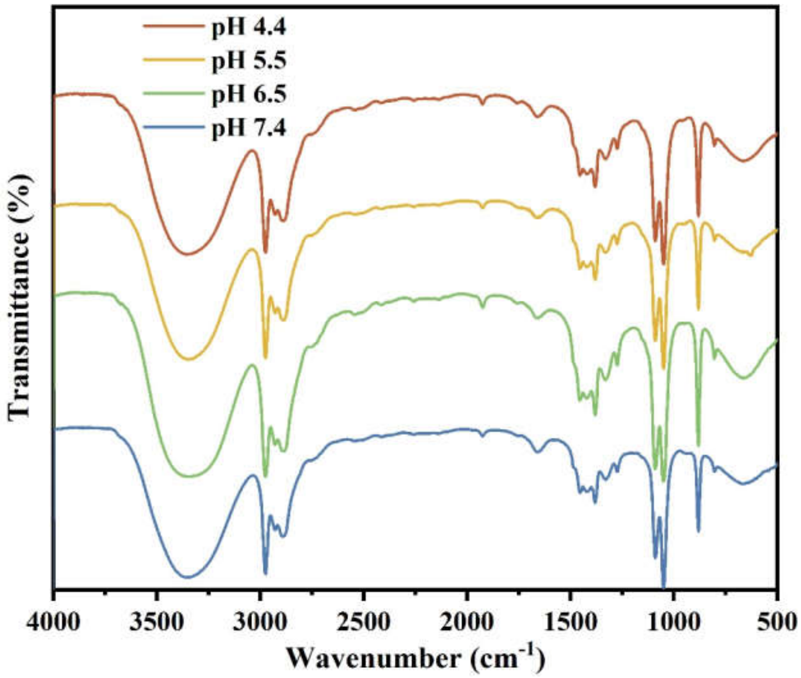

Figure S9. FTIR spectra of the CeO_2@PST nanomotors at different pH conditions.
